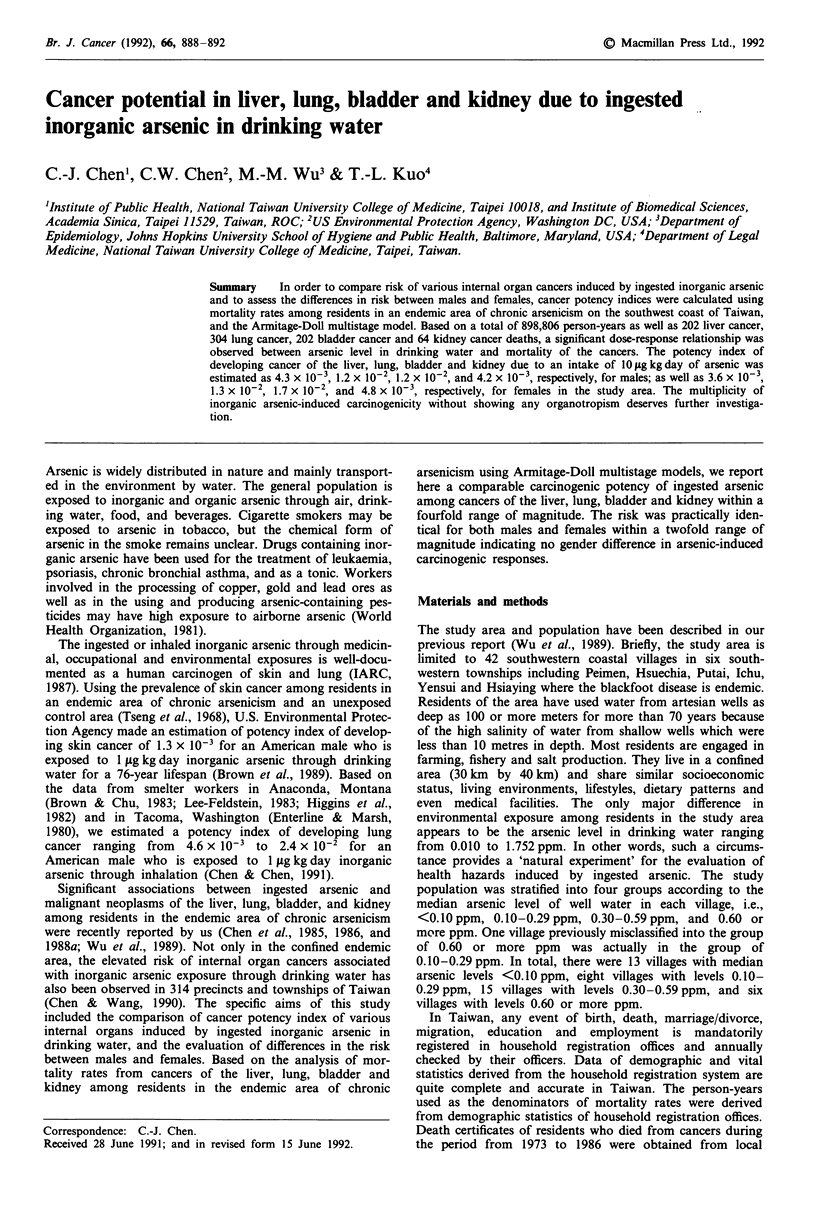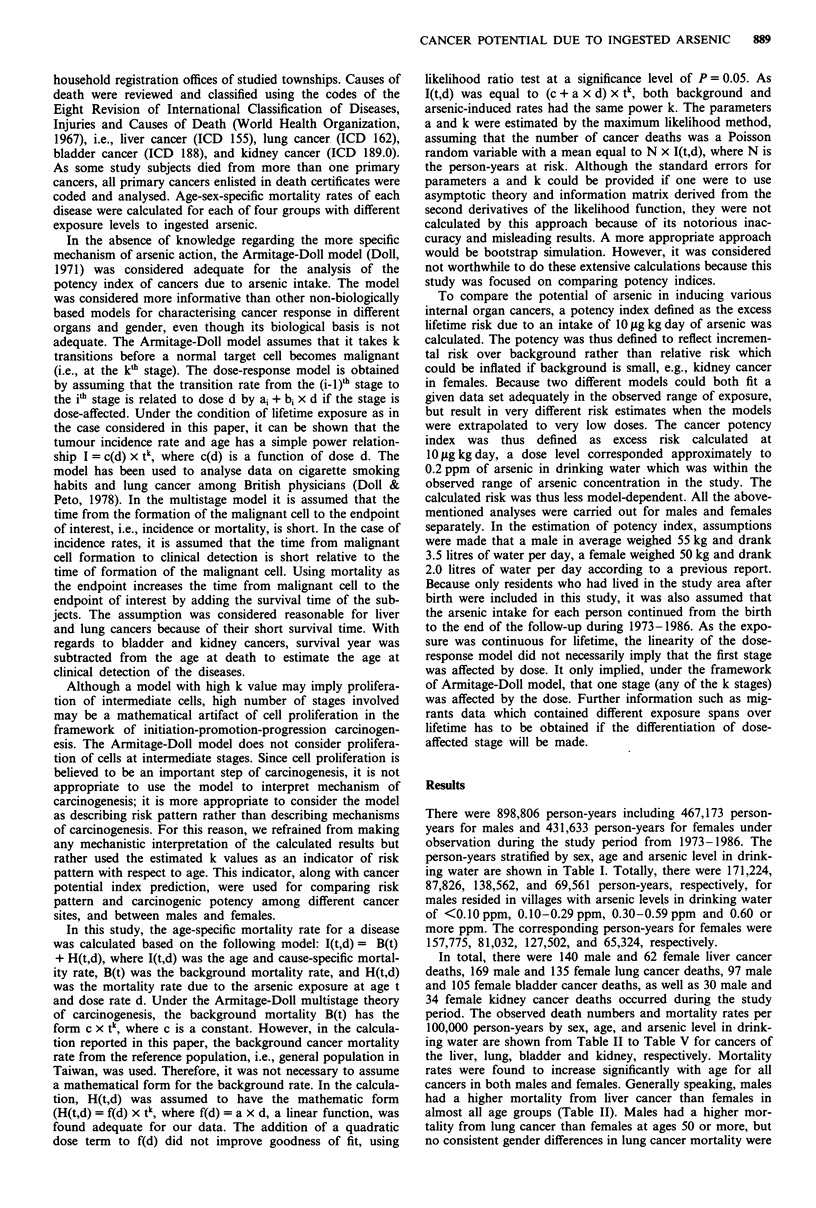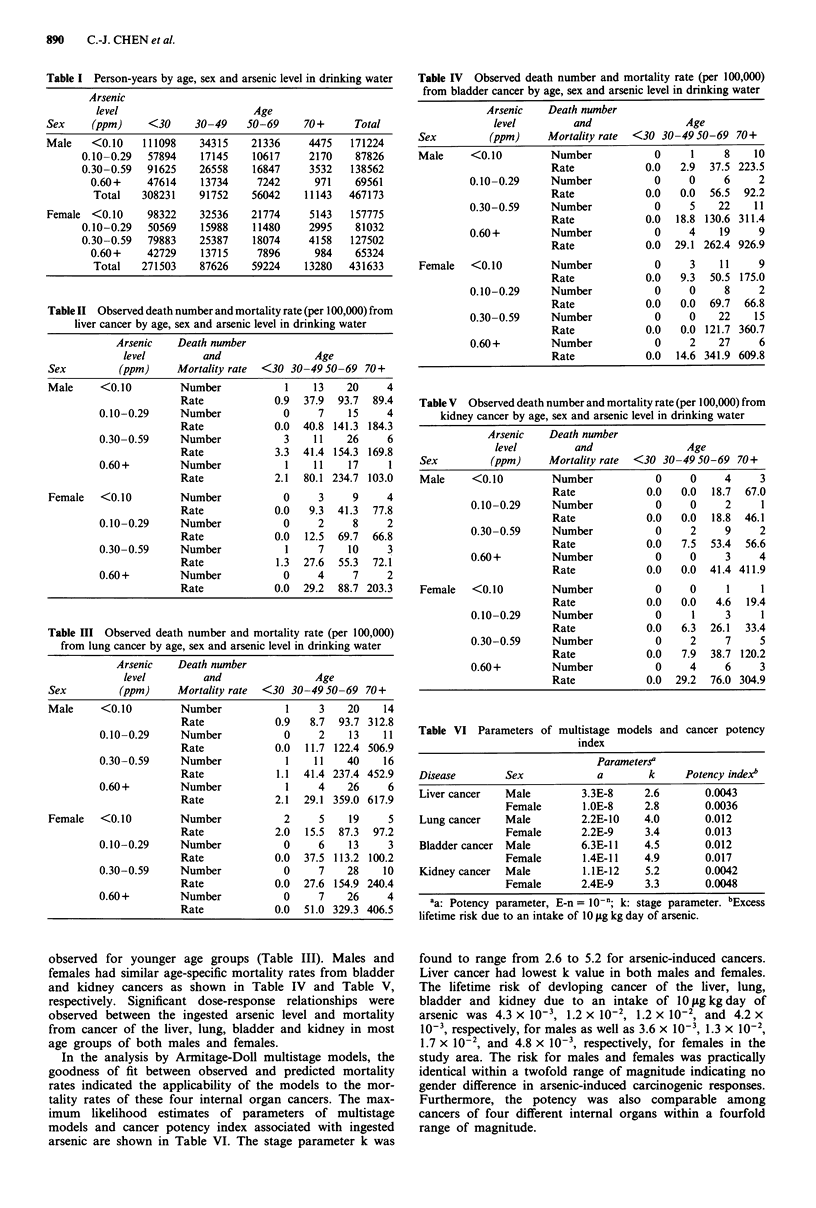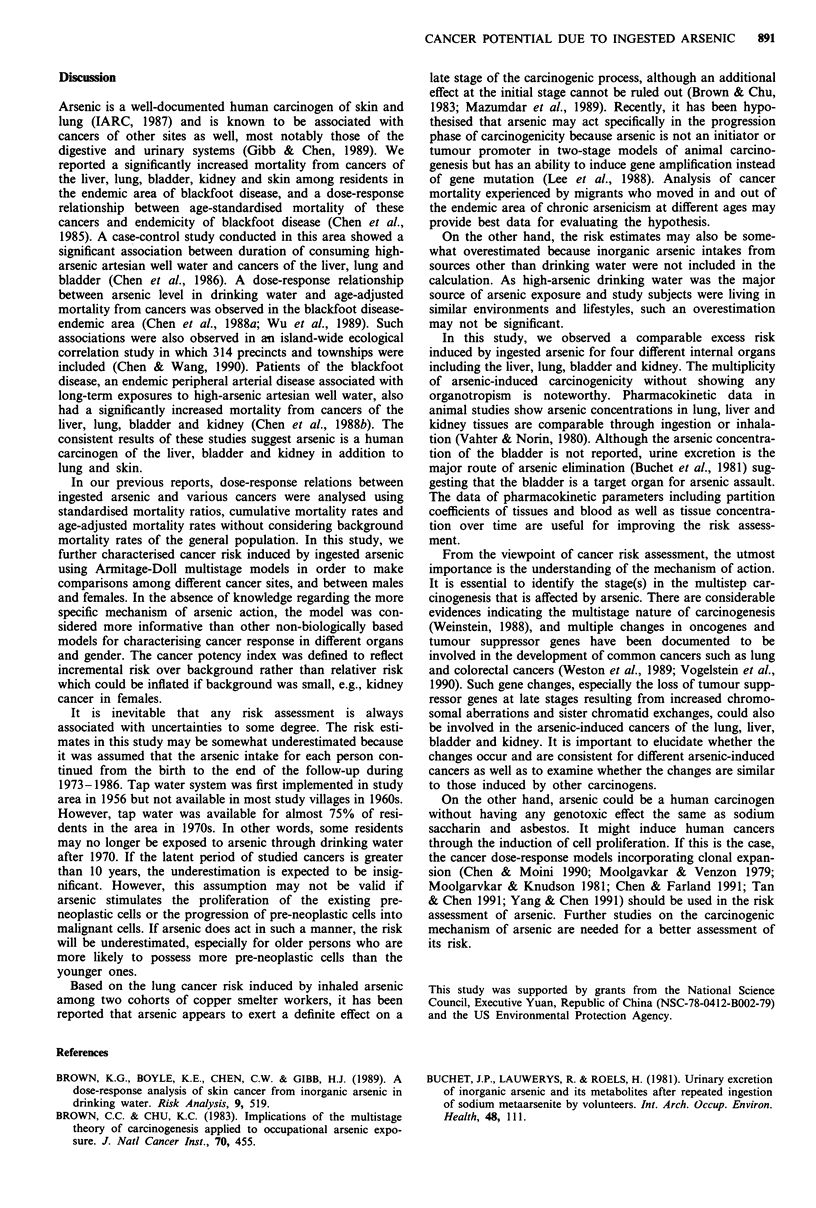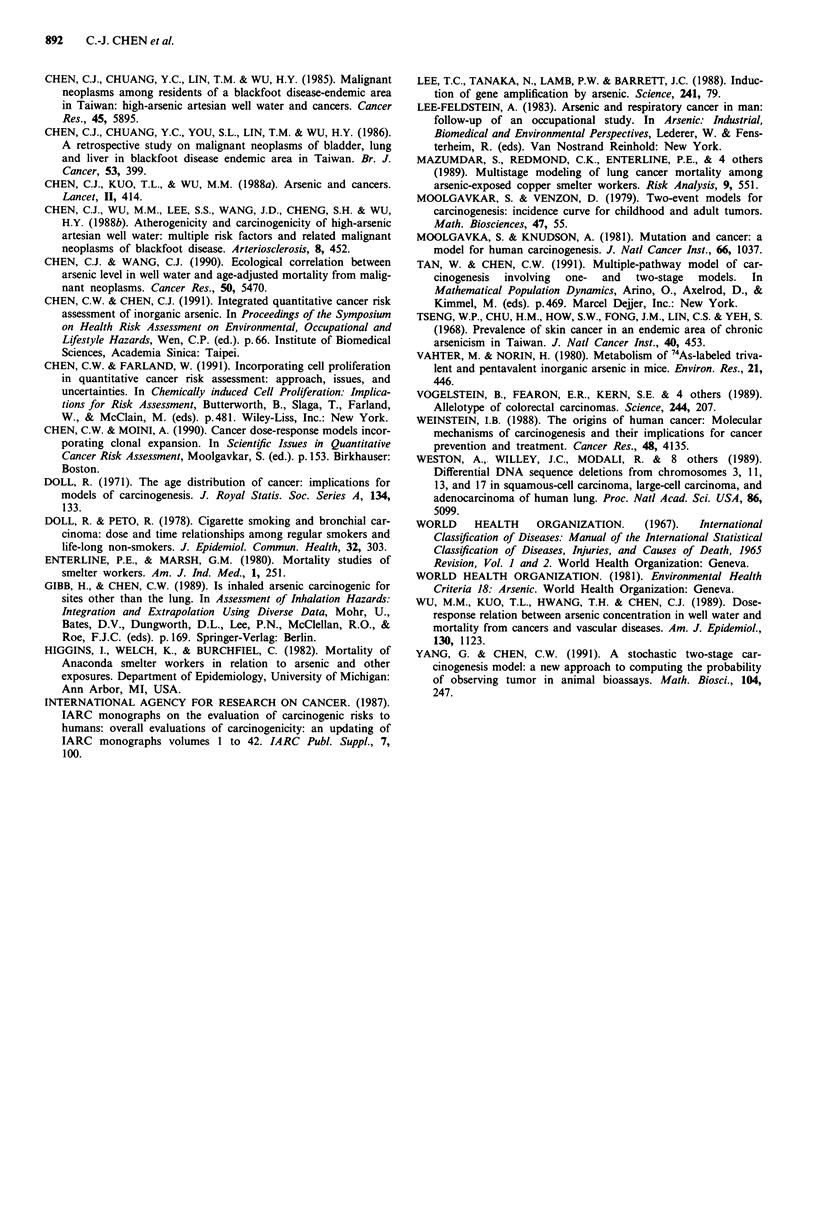# Cancer potential in liver, lung, bladder and kidney due to ingested inorganic arsenic in drinking water.

**DOI:** 10.1038/bjc.1992.380

**Published:** 1992-11

**Authors:** C. J. Chen, C. W. Chen, M. M. Wu, T. L. Kuo

**Affiliations:** Institute of Public Health, National Taiwan University College of Medicine, Taipei, ROC.

## Abstract

In order to compare risk of various internal organ cancers induced by ingested inorganic arsenic and to assess the differences in risk between males and females, cancer potency indices were calculated using mortality rates among residents in an endemic area of chronic arsenicism on the southwest coast of Taiwan, and the Armitage-Doll multistage model. Based on a total of 898,806 person-years as well as 202 liver cancer, 304 lung cancer, 202 bladder cancer and 64 kidney cancer deaths, a significant dose-response relationship was observed between arsenic level in drinking water and mortality of the cancers. The potency index of developing cancer of the liver, lung, bladder and kidney due to an intake of 10 micrograms kg day of arsenic was estimated as 4.3 x 10(-3), 1.2 x 10(-2), 1.2 x 10(-2), and 4.2 x 10(-3), respectively, for males; as well as 3.6 x 10(-3), 1.3 x 10(-2), 1.7 x 10(-2), and 4.8 x 10(-3), respectively, for females in the study area. The multiplicity of inorganic arsenic-induced carcinogenicity without showing any organotropism deserves further investigation.


*Br. J. Cancer* (1992), **66**, 888–892 © Macmillan Press Ltd., 1992

### Cancer potential in liver, lung, bladder and kidney due to ingested inorganic arsenic in drinking water

C.-J. Chen[1], C.W. Chen[2], M.-M. Wu[3] & T.-L. Kuo[4]

[1]*Institute of Public Health, National Taiwan University College of Medicine, Taipei 10018, and Institute of Biomedical Sciences, Academia Sinica, Taipei 11529, Taiwan, ROC;* [2]*US Environmental Protection Agency, Washington DC, USA;* [3]*Department of Epidemiology, Johns Hopkins University School of Hygiene and Public Health, Baltimore, Maryland, USA;* [4]*Department of Legal Medicine, National Taiwan University College of Medicine, Taipei, Taiwan.*

**Summary** In order to compare risk of various internal organ cancers induced by ingested inorganic arsenic and to assess the differences in risk between males and females, cancer potency indices were calculated using mortality rates among residents in an endemic area of chronic arsenicism on the southwest coast of Taiwan, and the Armitage-Doll multistage model. Based on a total of 898,806 person-years as well as 202 liver cancer, 304 lung cancer, 202 bladder cancer and 64 kidney cancer deaths, a significant dose-response relationship was observed between arsenic level in drinking water and mortality of the cancers. The potency index of developing cancer of the liver, lung, bladder and kidney due to an intake of 10 μg kg day of arsenic was estimated as $4.3 \times 10^{-3}$, $1.2 \times 10^{-2}$, $1.2 \times 10^{-2}$, and $4.2 \times 10^{-3}$, respectively, for males; as well as $3.6 \times 10^{-3}$, $1.3 \times 10^{-2}$, $1.7 \times 10^{-2}$, and $4.8 \times 10^{-3}$, respectively, for females in the study area. The multiplicity of inorganic arsenic-induced carcinogenicity without showing any organotropism deserves further investigation.

Arsenic is widely distributed in nature and mainly transported in the environment by water. The general population is exposed to inorganic and organic arsenic through air, drinking water, food, and beverages. Cigarette smokers may be exposed to arsenic in tobacco, but the chemical form of arsenic in the smoke remains unclear. Drugs containing inorganic arsenic have been used for the treatment of leukaemia, psoriasis, chronic bronchial asthma, and as a tonic. Workers involved in the processing of copper, gold and lead ores as well as in the using and producing arsenic-containing pesticides may have high exposure to airborne arsenic (World Health Organization, 1981).

The ingested or inhaled inorganic arsenic through medicinal, occupational and environmental exposures is well-documented as a human carcinogen of skin and lung (IARC, 1987). Using the prevalence of skin cancer among residents in an endemic area of chronic arsenicism and an unexposed control area (Tseng *et al.*, 1968), U.S. Environmental Protection Agency made an estimation of potency index of developing skin cancer of $1.3 \times 10^{-3}$ for an American male who is exposed to 1 μg kg day inorganic arsenic through drinking water for a 76-year lifespan (Brown *et al.*, 1989). Based on the data from smelter workers in Anaconda, Montana (Brown & Chu, 1983; Lee-Feldstein, 1983; Higgins *et al.*, 1982) and in Tacoma, Washington (Enterline & Marsh, 1980), we estimated a potency index of developing lung cancer ranging from $4.6 \times 10^{-3}$ to $2.4 \times 10^{-2}$ for an American male who is exposed to 1 μg kg day inorganic arsenic through inhalation (Chen & Chen, 1991).

Significant associations between ingested arsenic and malignant neoplasms of the liver, lung, bladder, and kidney among residents in the endemic area of chronic arsenicism were recently reported by us (Chen *et al.*, 1985, 1986, and 1988*a*; Wu *et al.*, 1989). Not only in the confined endemic area, the elevated risk of internal organ cancers associated with inorganic arsenic exposure through drinking water has also been observed in 314 precincts and townships of Taiwan (Chen & Wang, 1990). The specific aims of this study included the comparison of cancer potency index of various internal organs induced by ingested inorganic arsenic in drinking water, and the evaluation of differences in the risk between males and females. Based on the analysis of mortality rates from cancers of the liver, lung, bladder and kidney among residents in the endemic area of chronic arsenicism using Armitage-Doll multistage models, we report here a comparable carcinogenic potency of ingested arsenic among cancers of the liver, lung, bladder and kidney within a fourfold range of magnitude. The risk was practically identical for both males and females within a twofold range of magnitude indicating no gender difference in arsenic-induced carcinogenic responses.

### Materials and methods

The study area and population have been described in our previous report (Wu *et al.*, 1989). Briefly, the study area is limited to 42 southwestern coastal villages in six southwestern townships including Peimen, Hsuechia, Putai, Ichu, Yensui and Hsiaying where the blackfoot disease is endemic. Residents of the area have used water from artesian wells as deep as 100 or more meters for more than 70 years because of the high salinity of water from shallow wells which were less than 10 metres in depth. Most residents are engaged in farming, fishery and salt production. They live in a confined area (30 km by 40 km) and share similar socioeconomic status, living environments, lifestyles, dietary patterns and even medical facilities. The only major difference in environmental exposure among residents in the study area appears to be the arsenic level in drinking water ranging from 0.010 to 1.752 ppm. In other words, such a circumstance provides a 'natural experiment' for the evaluation of health hazards induced by ingested arsenic. The study population was stratified into four groups according to the median arsenic level of well water in each village, i.e., <0.10 ppm, 0.10–0.29 ppm, 0.30–0.59 ppm, and 0.60 or more ppm. One village previously misclassified into the group of 0.60 or more ppm was actually in the group of 0.10–0.29 ppm. In total, there were 13 villages with median arsenic levels <0.10 ppm, eight villages with levels 0.10–0.29 ppm, 15 villages with levels 0.30–0.59 ppm, and six villages with levels 0.60 or more ppm.

In Taiwan, any event of birth, death, marriage/divorce, migration, education and employment is mandatorily registered in household registration offices and annually checked by their officers. Data of demographic and vital statistics derived from the household registration system are quite complete and accurate in Taiwan. The person-years used as the denominators of mortality rates were derived from demographic statistics of household registration offices. Death certificates of residents who died from cancers during the period from 1973 to 1986 were obtained from local

Correspondence: C.-J. Chen.
Received 28 June 1991; and in revised form 15 June 1992.

household registration offices of studied townships. Causes of death were reviewed and classified using the codes of the Eight Revision of International Classification of Diseases, Injuries and Causes of Death (World Health Organization, 1967), i.e., liver cancer (ICD 155), lung cancer (ICD 162), bladder cancer (ICD 188), and kidney cancer (ICD 189.0). As some study subjects died from more than one primary cancers, all primary cancers enlisted in death certificates were coded and analysed. Age-sex-specific mortality rates of each disease were calculated for each of four groups with different exposure levels to ingested arsenic.

In the absence of knowledge regarding the more specific mechanism of arsenic action, the Armitage-Doll model (Doll, 1971) was considered adequate for the analysis of the potency index of cancers due to arsenic intake. The model was considered more informative than other non-biologically based models for characterising cancer response in different organs and gender, even though its biological basis is not adequate. The Armitage-Doll model assumes that it takes k transitions before a normal target cell becomes malignant (i.e., at the $k^{th}$ stage). The dose-response model is obtained by assuming that the transition rate from the $(i-1)^{th}$ stage to the $i^{th}$ stage is related to dose d by $a_i + b_i \times d$ if the stage is dose-affected. Under the condition of lifetime exposure as in the case considered in this paper, it can be shown that the tumour incidence rate and age has a simple power relationship $I = c(d) \times t^k$, where c(d) is a function of dose d. The model has been used to analyse data on cigarette smoking habits and lung cancer among British physicians (Doll & Peto, 1978). In the multistage model it is assumed that the time from the formation of the malignant cell to the endpoint of interest, i.e., incidence or mortality, is short. In the case of incidence rates, it is assumed that the time from malignant cell formation to clinical detection is short relative to the time of formation of the malignant cell. Using mortality as the endpoint increases the time from malignant cell to the endpoint of interest by adding the survival time of the subjects. The assumption was considered reasonable for liver and lung cancers because of their short survival time. With regards to bladder and kidney cancers, survival year was subtracted from the age at death to estimate the age at clinical detection of the diseases.

Although a model with high k value may imply proliferation of intermediate cells, high number of stages involved may be a mathematical artifact of cell proliferation in the framework of initiation-promotion-progression carcinogenesis. The Armitage-Doll model does not consider proliferation of cells at intermediate stages. Since cell proliferation is believed to be an important step of carcinogenesis, it is not appropriate to use the model to interpret mechanism of carcinogenesis; it is more appropriate to consider the model as describing risk pattern rather than describing mechanisms of carcinogenesis. For this reason, we refrained from making any mechanistic interpretation of the calculated results but rather used the estimated k values as an indicator of risk pattern with respect to age. This indicator, along with cancer potential index prediction, were used for comparing risk pattern and carcinogenic potency among different cancer sites, and between males and females.

In this study, the age-specific mortality rate for a disease was calculated based on the following model: $I(t,d) = B(t) + H(t,d)$, where I(t,d) was the age and cause-specific mortality rate, B(t) was the background mortality rate, and H(t,d) was the mortality rate due to the arsenic exposure at age t and dose rate d. Under the Armitage-Doll multistage theory of carcinogenesis, the background mortality B(t) has the form $c \times t^k$, where c is a constant. However, in the calculation reported in this paper, the background cancer mortality rate from the reference population, i.e., general population in Taiwan, was used. Therefore, it was not necessary to assume a mathematical form for the background rate. In the calculation, H(t,d) was assumed to have the mathematic form $(H(t,d) = f(d) \times t^k$, where $f(d) = a \times d$, a linear function, was found adequate for our data. The addition of a quadratic dose term to f(d) did not improve goodness of fit, using likelihood ratio test at a significance level of $P = 0.05$. As I(t,d) was equal to $(c + a \times d) \times t^k$, both background and arsenic-induced rates had the same power k. The parameters a and k were estimated by the maximum likelihood method, assuming that the number of cancer deaths was a Poisson random variable with a mean equal to $N \times I(t,d)$, where N is the person-years at risk. Although the standard errors for parameters a and k could be provided if one were to use asymptotic theory and information matrix derived from the second derivatives of the likelihood function, they were not calculated by this approach because of its notorious inaccuracy and misleading results. A more appropriate approach would be bootstrap simulation. However, it was considered not worthwhile to do these extensive calculations because this study was focused on comparing potency indices.

To compare the potential of arsenic in inducing various internal organ cancers, a potency index defined as the excess lifetime risk due to an intake of 10 μg kg day of arsenic was calculated. The potency was thus defined to reflect incremental risk over background rather than relative risk which could be inflated if background is small, e.g., kidney cancer in females. Because two different models could both fit a given data set adequately in the observed range of exposure, but result in very different risk estimates when the models were extrapolated to very low doses. The cancer potency index was thus defined as excess risk calculated at 10 μg kg day, a dose level corresponded approximately to 0.2 ppm of arsenic in drinking water which was within the observed range of arsenic concentration in the study. The calculated risk was thus less model-dependent. All the abovementioned analyses were carried out for males and females separately. In the estimation of potency index, assumptions were made that a male in average weighed 55 kg and drank 3.5 litres of water per day, a female weighed 50 kg and drank 2.0 litres of water per day according to a previous report. Because only residents who had lived in the study area after birth were included in this study, it was also assumed that the arsenic intake for each person continued from the birth to the end of the follow-up during 1973–1986. As the exposure was continuous for lifetime, the linearity of the dose-response model did not necessarily imply that the first stage was affected by dose. It only implied, under the framework of Armitage-Doll model, that one stage (any of the k stages) was affected by the dose. Further information such as migrants data which contained different exposure spans over lifetime has to be obtained if the differentiation of dose-affected stage will be made.

### Results

There were 898,806 person-years including 467,173 person-years for males and 431,633 person-years for females under observation during the study period from 1973–1986. The person-years stratified by sex, age and arsenic level in drinking water are shown in Table I. Totally, there were 171,224, 87,826, 138,562, and 69,561 person-years, respectively, for males resided in villages with arsenic levels in drinking water of <0.10 ppm, 0.10–0.29 ppm, 0.30–0.59 ppm and 0.60 or more ppm. The corresponding person-years for females were 157,775, 81,032, 127,502, and 65,324, respectively.

In total, there were 140 male and 62 female liver cancer deaths, 169 male and 135 female lung cancer deaths, 97 male and 105 female bladder cancer deaths, as well as 30 male and 34 female kidney cancer deaths occurred during the study period. The observed death numbers and mortality rates per 100,000 person-years by sex, age, and arsenic level in drinking water are shown from Table II to Table V for cancers of the liver, lung, bladder and kidney, respectively. Mortality rates were found to increase significantly with age for all cancers in both males and females. Generally speaking, males had a higher mortality from liver cancer than females in almost all age groups (Table II). Males had a higher mortality from lung cancer than females at ages 50 or more, but no consistent gender differences in lung cancer mortality were

**Table I** Person-years by age, sex and arsenic level in drinking water

| Sex | Arsenic level (ppm) | Age <30 | 30–49 | 50–69 | 70+ | Total |
|---|---|---|---|---|---|---|
| Male | <0.10 | 111098 | 34315 | 21336 | 4475 | 171224 |
| | 0.10–0.29 | 57894 | 17145 | 10617 | 2170 | 87826 |
| | 0.30–0.59 | 91625 | 26558 | 16847 | 3532 | 138562 |
| | 0.60+ | 47614 | 13734 | 7242 | 971 | 69561 |
| | Total | 308231 | 91752 | 56042 | 11143 | 467173 |
| Female | <0.10 | 98322 | 32536 | 21774 | 5143 | 157775 |
| | 0.10–0.29 | 50569 | 15988 | 11480 | 2995 | 81032 |
| | 0.30–0.59 | 79883 | 25387 | 18074 | 4158 | 127502 |
| | 0.60+ | 42729 | 13715 | 7896 | 984 | 65324 |
| | Total | 271503 | 87626 | 59224 | 13280 | 431633 |

**Table II** Observed death number and mortality rate (per 100,000) from liver cancer by age, sex and arsenic level in drinking water

| Sex | Arsenic level (ppm) | Death number and Mortality rate | Age <30 | 30–49 | 50–69 | 70+ |
|---|---|---|---|---|---|---|
| Male | <0.10 | Number | 1 | 13 | 20 | 4 |
| | | Rate | 0.9 | 37.9 | 93.7 | 89.4 |
| | 0.10–0.29 | Number | 0 | 7 | 15 | 4 |
| | | Rate | 0.0 | 40.8 | 141.3 | 184.3 |
| | 0.30–0.59 | Number | 3 | 11 | 26 | 6 |
| | | Rate | 3.3 | 41.4 | 154.3 | 169.8 |
| | 0.60+ | Number | 1 | 11 | 17 | 1 |
| | | Rate | 2.1 | 80.1 | 234.7 | 103.0 |
| Female | <0.10 | Number | 0 | 3 | 9 | 4 |
| | | Rate | 0.0 | 9.3 | 41.3 | 77.8 |
| | 0.10–0.29 | Number | 0 | 2 | 8 | 2 |
| | | Rate | 0.0 | 12.5 | 69.7 | 66.8 |
| | 0.30–0.59 | Number | 1 | 7 | 10 | 3 |
| | | Rate | 1.3 | 27.6 | 55.3 | 72.1 |
| | 0.60+ | Number | 0 | 4 | 7 | 2 |
| | | Rate | 0.0 | 29.2 | 88.7 | 203.3 |

**Table III** Observed death number and mortality rate (per 100,000) from lung cancer by age, sex and arsenic level in drinking water

| Sex | Arsenic level (ppm) | Death number and Mortality rate | Age <30 | 30–49 | 50–69 | 70+ |
|---|---|---|---|---|---|---|
| Male | <0.10 | Number | 1 | 3 | 20 | 14 |
| | | Rate | 0.9 | 8.7 | 93.7 | 312.8 |
| | 0.10–0.29 | Number | 0 | 2 | 13 | 11 |
| | | Rate | 0.0 | 11.7 | 122.4 | 506.9 |
| | 0.30–0.59 | Number | 1 | 11 | 40 | 16 |
| | | Rate | 1.1 | 41.4 | 237.4 | 452.9 |
| | 0.60+ | Number | 1 | 4 | 26 | 6 |
| | | Rate | 2.1 | 29.1 | 359.0 | 617.9 |
| Female | <0.10 | Number | 2 | 5 | 19 | 5 |
| | | Rate | 2.0 | 15.5 | 87.3 | 97.2 |
| | 0.10–0.29 | Number | 0 | 6 | 13 | 3 |
| | | Rate | 0.0 | 37.5 | 113.2 | 100.2 |
| | 0.30–0.59 | Number | 0 | 7 | 28 | 10 |
| | | Rate | 0.0 | 27.6 | 154.9 | 240.4 |
| | 0.60+ | Number | 0 | 7 | 26 | 4 |
| | | Rate | 0.0 | 51.0 | 329.3 | 406.5 |

**Table IV** Observed death number and mortality rate (per 100,000) from bladder cancer by age, sex and arsenic level in drinking water

| Sex | Arsenic level (ppm) | Death number and Mortality rate | Age <30 | 30–49 | 50–69 | 70+ |
|---|---|---|---|---|---|---|
| Male | <0.10 | Number | 0 | 1 | 8 | 10 |
| | | Rate | 0.0 | 2.9 | 37.5 | 223.5 |
| | 0.10–0.29 | Number | 0 | 0 | 6 | 2 |
| | | Rate | 0.0 | 0.0 | 56.5 | 92.2 |
| | 0.30–0.59 | Number | 0 | 5 | 22 | 11 |
| | | Rate | 0.0 | 18.8 | 130.6 | 311.4 |
| | 0.60+ | Number | 0 | 4 | 19 | 9 |
| | | Rate | 0.0 | 29.1 | 262.4 | 926.9 |
| Female | <0.10 | Number | 0 | 3 | 11 | 9 |
| | | Rate | 0.0 | 9.3 | 50.5 | 175.0 |
| | 0.10–0.29 | Number | 0 | 0 | 8 | 2 |
| | | Rate | 0.0 | 0.0 | 69.7 | 66.8 |
| | 0.30–0.59 | Number | 0 | 0 | 22 | 15 |
| | | Rate | 0.0 | 0.0 | 121.7 | 360.7 |
| | 0.60+ | Number | 0 | 2 | 27 | 6 |
| | | Rate | 0.0 | 14.6 | 341.9 | 609.8 |

**Table V** Observed death number and mortality rate (per 100,000) from kidney cancer by age, sex and arsenic level in drinking water

| Sex | Arsenic level (ppm) | Death number and Mortality rate | Age <30 | 30–49 | 50–69 | 70+ |
|---|---|---|---|---|---|---|
| Male | <0.10 | Number | 0 | 0 | 4 | 3 |
| | | Rate | 0.0 | 0.0 | 18.7 | 67.0 |
| | 0.10–0.29 | Number | 0 | 0 | 2 | 1 |
| | | Rate | 0.0 | 0.0 | 18.8 | 46.1 |
| | 0.30–0.59 | Number | 0 | 2 | 9 | 2 |
| | | Rate | 0.0 | 7.5 | 53.4 | 56.6 |
| | 0.60+ | Number | 0 | 0 | 3 | 4 |
| | | Rate | 0.0 | 0.0 | 41.4 | 411.9 |
| Female | <0.10 | Number | 0 | 0 | 1 | 1 |
| | | Rate | 0.0 | 0.0 | 4.6 | 19.4 |
| | 0.10–0.29 | Number | 0 | 1 | 3 | 1 |
| | | Rate | 0.0 | 6.3 | 26.1 | 33.4 |
| | 0.30–0.59 | Number | 0 | 2 | 7 | 5 |
| | | Rate | 0.0 | 7.9 | 38.7 | 120.2 |
| | 0.60+ | Number | 0 | 4 | 6 | 3 |
| | | Rate | 0.0 | 29.2 | 76.0 | 304.9 |

**Table VI** Parameters of multistage models and cancer potency index

| Disease | Sex | Parameters[a] a | k | Potency index[b] |
|---|---|---|---|---|
| Liver cancer | Male | 3.3E-8 | 2.6 | 0.0043 |
| | Female | 1.0E-8 | 2.8 | 0.0036 |
| Lung cancer | Male | 2.2E-10 | 4.0 | 0.012 |
| | Female | 2.2E-9 | 3.4 | 0.013 |
| Bladder cancer | Male | 6.3E-11 | 4.5 | 0.012 |
| | Female | 1.4E-11 | 4.9 | 0.017 |
| Kidney cancer | Male | 1.1E-12 | 5.2 | 0.0042 |
| | Female | 2.4E-9 | 3.3 | 0.0048 |

[a]a: Potency parameter, E-n = $10^{-n}$; k: stage parameter. [b]Excess lifetime risk due to an intake of 10 μg kg day of arsenic.

observed for younger age groups (Table III). Males and females had similar age-specific mortality rates from bladder and kidney cancers as shown in Table IV and Table V, respectively. Significant dose-response relationships were observed between the ingested arsenic level and mortality from cancer of the liver, lung, bladder and kidney in most age groups of both males and females.

In the analysis by Armitage-Doll multistage models, the goodness of fit between observed and predicted mortality rates indicated the applicability of the models to the mortality rates of these four internal organ cancers. The maximum likelihood estimates of parameters of multistage models and cancer potency index associated with ingested arsenic are shown in Table VI. The stage parameter k was found to range from 2.6 to 5.2 for arsenic-induced cancers. Liver cancer had lowest k value in both males and females. The lifetime risk of devloping cancer of the liver, lung, bladder and kidney due to an intake of 10 μg kg day of arsenic was $4.3 \times 10^{-3}$, $1.2 \times 10^{-2}$, $1.2 \times 10^{-2}$, and $4.2 \times 10^{-3}$, respectively, for males as well as $3.6 \times 10^{-3}$, $1.3 \times 10^{-2}$, $1.7 \times 10^{-2}$, and $4.8 \times 10^{-3}$, respectively, for females in the study area. The risk for males and females was practically identical within a twofold range of magnitude indicating no gender difference in arsenic-induced carcinogenic responses. Furthermore, the potency was also comparable among cancers of four different internal organs within a fourfold range of magnitude.

**Discussion**

Arsenic is a well-documented human carcinogen of skin and lung (IARC, 1987) and is known to be associated with cancers of other sites as well, most notably those of the digestive and urinary systems (Gibb & Chen, 1989). We reported a significantly increased mortality from cancers of the liver, lung, bladder, kidney and skin among residents in the endemic area of blackfoot disease, and a dose-response relationship between age-standardised mortality of these cancers and endemicity of blackfoot disease (Chen *et al.*, 1985). A case-control study conducted in this area showed a significant association between duration of consuming high-arsenic artesian well water and cancers of the liver, lung and bladder (Chen *et al.*, 1986). A dose-response relationship between arsenic level in drinking water and age-adjusted mortality from cancers was observed in the blackfoot disease-endemic area (Chen *et al.*, 1988*a*; Wu *et al.*, 1989). Such associations were also observed in an island-wide ecological correlation study in which 314 precincts and townships were included (Chen & Wang, 1990). Patients of the blackfoot disease, an endemic peripheral arterial disease associated with long-term exposures to high-arsenic artesian well water, also had a significantly increased mortality from cancers of the liver, lung, bladder and kidney (Chen *et al.*, 1988*b*). The consistent results of these studies suggest arsenic is a human carcinogen of the liver, bladder and kidney in addition to lung and skin.

In our previous reports, dose-response relations between ingested arsenic and various cancers were analysed using standardised mortality ratios, cumulative mortality rates and age-adjusted mortality rates without considering background mortality rates of the general population. In this study, we further characterised cancer risk induced by ingested arsenic using Armitage-Doll multistage models in order to make comparisons among different cancer sites, and between males and females. In the absence of knowledge regarding the more specific mechanism of arsenic action, the model was considered more informative than other non-biologically based models for characterising cancer response in different organs and gender. The cancer potency index was defined to reflect incremental risk over background rather than relativer risk which could be inflated if background was small, e.g., kidney cancer in females.

It is inevitable that any risk assessment is always associated with uncertainties to some degree. The risk estimates in this study may be somewhat underestimated because it was assumed that the arsenic intake for each person continued from the birth to the end of the follow-up during 1973–1986. Tap water system was first implemented in study area in 1956 but not available in most study villages in 1960s. However, tap water was available for almost 75% of residents in the area in 1970s. In other words, some residents may no longer be exposed to arsenic through drinking water after 1970. If the latent period of studied cancers is greater than 10 years, the underestimation is expected to be insignificant. However, this assumption may not be valid if arsenic stimulates the proliferation of the existing pre-neoplastic cells or the progression of pre-neoplastic cells into malignant cells. If arsenic does act in such a manner, the risk will be underestimated, especially for older persons who are more likely to possess more pre-neoplastic cells than the younger ones.

Based on the lung cancer risk induced by inhaled arsenic among two cohorts of copper smelter workers, it has been reported that arsenic appears to exert a definite effect on a late stage of the carcinogenic process, although an additional effect at the initial stage cannot be ruled out (Brown & Chu, 1983; Mazumdar *et al.*, 1989). Recently, it has been hypothesised that arsenic may act specifically in the progression phase of carcinogenicity because arsenic is not an initiator or tumour promoter in two-stage models of animal carcinogenesis but has an ability to induce gene amplification instead of gene mutation (Lee *et al.*, 1988). Analysis of cancer mortality experienced by migrants who moved in and out of the endemic area of chronic arsenicism at different ages may provide best data for evaluating the hypothesis.

On the other hand, the risk estimates may also be somewhat overestimated because inorganic arsenic intakes from sources other than drinking water were not included in the calculation. As high-arsenic drinking water was the major source of arsenic exposure and study subjects were living in similar environments and lifestyles, such an overestimation may not be significant.

In this study, we observed a comparable excess risk induced by ingested arsenic for four different internal organs including the liver, lung, bladder and kidney. The multiplicity of arsenic-induced carcinogenicity without showing any organotropism is noteworthy. Pharmacokinetic data in animal studies show arsenic concentrations in lung, liver and kidney tissues are comparable through ingestion or inhalation (Vahter & Norin, 1980). Although the arsenic concentration of the bladder is not reported, urine excretion is the major route of arsenic elimination (Buchet *et al.*, 1981) suggesting that the bladder is a target organ for arsenic assault. The data of pharmacokinetic parameters including partition coefficients of tissues and blood as well as tissue concentration over time are useful for improving the risk assessment.

From the viewpoint of cancer risk assessment, the utmost importance is the understanding of the mechanism of action. It is essential to identify the stage(s) in the multistep carcinogenesis that is affected by arsenic. There are considerable evidences indicating the multistage nature of carcinogenesis (Weinstein, 1988), and multiple changes in oncogenes and tumour suppressor genes have been documented to be involved in the development of common cancers such as lung and colorectal cancers (Weston *et al.*, 1989; Vogelstein *et al.*, 1990). Such gene changes, especially the loss of tumour suppressor genes at late stages resulting from increased chromosomal aberrations and sister chromatid exchanges, could also be involved in the arsenic-induced cancers of the lung, liver, bladder and kidney. It is important to elucidate whether the changes occur and are consistent for different arsenic-induced cancers as well as to examine whether the changes are similar to those induced by other carcinogens.

On the other hand, arsenic could be a human carcinogen without having any genotoxic effect the same as sodium saccharin and asbestos. It might induce human cancers through the induction of cell proliferation. If this is the case, the cancer dose-response models incorporating clonal expansion (Chen & Moini 1990; Moolgavkar & Venzon 1979; Moolgarvkar & Knudson 1981; Chen & Farland 1991; Tan & Chen 1991; Yang & Chen 1991) should be used in the risk assessment of arsenic. Further studies on the carcinogenic mechanism of arsenic are needed for a better assessment of its risk.

This study was supported by grants from the National Science Council, Executive Yuan, Republic of China (NSC-78-0412-B002-79) and the US Environmental Protection Agency.

**References**

BROWN, K.G., BOYLE, K.E., CHEN, C.W. & GIBB, H.J. (1989). A dose-response analysis of skin cancer from inorganic arsenic in drinking water. *Risk Analysis*, **9**, 519.

BROWN, C.C. & CHU, K.C. (1983). Implications of the multistage theory of carcinogenesis applied to occupational arsenic exposure. *J. Natl Cancer Inst.*, **70**, 455.

BUCHET, J.P., LAUWERYS, R. & ROELS, H. (1981). Urinary excretion of inorganic arsenic and its metabolites after repeated ingestion of sodium metaarsenite by volunteers. *Int. Arch. Occup. Environ. Health*, **48**, 111.

CHEN, C.J., CHUANG, Y.C., LIN, T.M. & WU, H.Y. (1985). Malignant neoplasms among residents of a blackfoot disease-endemic area in Taiwan: high-arsenic artesian well water and cancers. *Cancer Res.*, **45**, 5895.

CHEN, C.J., CHUANG, Y.C., YOU, S.L., LIN, T.M. & WU, H.Y. (1986). A retrospective study on malignant neoplasms of bladder, lung and liver in blackfoot disease endemic area in Taiwan. *Br. J. Cancer*, **53**, 399.

CHEN, C.J., KUO, T.L., & WU, M.M. (1988*a*). Arsenic and cancers. *Lancet*, **II**, 414.

CHEN, C.J., WU, M.M., LEE, S.S., WANG, J.D., CHENG, S.H. & WU, H.Y. (1988*b*). Atherogenicity and carcinogenicity of high-arsenic artesian well water: multiple risk factors and related malignant neoplasms of blackfoot disease. *Arteriosclerosis*, **8**, 452.

CHEN, C.J. & WANG, C.J. (1990). Ecological correlation between arsenic level in well water and age-adjusted mortality from malignant neoplasms. *Cancer Res.*, **50**, 5470.

CHEN, C.W. & CHEN, C.J. (1991). Integrated quantitative cancer risk assessment of inorganic arsenic. In *Proceedings of the Symposium on Health Risk Assessment on Environmental, Occupational and Lifestyle Hazards*, Wen, C.P. (ed.). p.66. Institute of Biomedical Sciences, Academia Sinica: Taipei.

CHEN, C.W. & FARLAND, W. (1991). Incorporating cell proliferation in quantitative cancer risk assessment: approach, issues, and uncertainties. In *Chemically induced Cell Proliferation: Implications for Risk Assessment*, Butterworth, B., Slaga, T., Farland, W., & McClain, M. (eds). p.481. Wiley-Liss, Inc.: New York.

CHEN, C.W. & MOINI, A. (1990). Cancer dose-response models incorporating clonal expansion. In *Scientific Issues in Quantitative Cancer Risk Assessment*, Moolgavkar, S. (ed.). p.153. Birkhauser: Boston.

DOLL, R. (1971). The age distribution of cancer: implications for models of carcinogenesis. *J. Royal Statis. Soc. Series A*, **134**, 133.

DOLL, R. & PETO, R. (1978). Cigarette smoking and bronchial carcinoma: dose and time relationships among regular smokers and life-long non-smokers. *J. Epidemiol. Commun. Health*, **32**, 303.

ENTERLINE, P.E., & MARSH, G.M. (1980). Mortality studies of smelter workers. *Am. J. Ind. Med.*, **1**, 251.

GIBB, H., & CHEN, C.W. (1989). Is inhaled arsenic carcinogenic for sites other than the lung. In *Assessment of Inhalation Hazards: Integration and Extrapolation Using Diverse Data*, Mohr, U., Bates, D.V., Dungworth, D.L., Lee, P.N., McClellan, R.O., & Roe, F.J.C. (eds). p.169. Springer-Verlag: Berlin.

HIGGINS, I., WELCH, K., & BURCHFIEL, C. (1982). Mortality of Anaconda smelter workers in relation to arsenic and other exposures. Department of Epidemiology, University of Michigan: Ann Arbor, MI, USA.

INTERNATIONAL AGENCY FOR RESEARCH ON CANCER. (1987). IARC monographs on the evaluation of carcinogenic risks to humans: overall evaluations of carcinogenicity: an updating of IARC monographs volumes 1 to 42. *IARC Publ. Suppl.*, **7**, 100.

LEE, T.C., TANAKA, N., LAMB, P.W. & BARRETT, J.C. (1988). Induction of gene amplification by arsenic. *Science*, **241**, 79.

LEE-FELDSTEIN, A. (1983). Arsenic and respiratory cancer in man: follow-up of an occupational study. In *Arsenic: Industrial, Biomedical and Environmental Perspectives*, Lederer, W. & Fensterheim, R. (eds). Van Nostrand Reinhold: New York.

MAZUMDAR, S., REDMOND, C.K., ENTERLINE, P.E., & 4 others (1989). Multistage modeling of lung cancer mortality among arsenic-exposed copper smelter workers. *Risk Analysis*, **9**, 551.

MOOLGAVKAR, S. & VENZON, D. (1979). Two-event models for carcinogenesis: incidence curve for childhood and adult tumors. *Math. Biosciences*, **47**, 55.

MOOLGAVKA, S. & KNUDSON, A. (1981). Mutation and cancer: a model for human carcinogenesis. *J. Natl Cancer Inst.*, **66**, 1037.

TAN, W. & CHEN, C.W. (1991). Multiple-pathway model of carcinogenesis involving one- and two-stage models. In *Mathematical Population Dynamics*, Arino, O., Axelrod, D., & Kimmel, M. (eds). p.469. Marcel Dejjer, Inc.: New York.

TSENG, W.P., CHU, H.M., HOW, S.W., FONG, J.M., LIN, C.S. & YEH, S. (1968). Prevalence of skin cancer in an endemic area of chronic arsenicism in Taiwan. *J. Natl Cancer Inst.*, **40**, 453.

VAHTER, M. & NORIN, H. (1980). Metabolism of $^{74}$As-labeled trivalent and pentavalent inorganic arsenic in mice. *Environ. Res.*, **21**, 446.

VOGELSTEIN, B., FEARON, E.R., KERN, S.E. & 4 others (1989). Allelotype of colorectal carcinomas. *Science*, **244**, 207.

WEINSTEIN, I.B. (1988). The origins of human cancer: Molecular mechanisms of carcinogenesis and their implications for cancer prevention and treatment. *Cancer Res.*, **48**, 4135.

WESTON, A., WILLEY, J.C., MODALI, R. & 8 others (1989). Differential DNA sequence deletions from chromosomes 3, 11, 13, and 17 in squamous-cell carcinoma, large-cell carcinoma, and adenocarcinoma of human lung. *Proc. Natl Acad. Sci. USA*, **86**, 5099.

WORLD HEALTH ORGANIZATION. (1967). *International Classification of Diseases: Manual of the International Statistical Classification of Diseases, Injuries, and Causes of Death, 1965 Revision, Vol. 1 and 2.* World Health Organization: Geneva.

WORLD HEALTH ORGANIZATION. (1981). *Environmental Health Criteria 18: Arsenic.* World Health Organization: Geneva.

WU, M.M., KUO, T.L., HWANG, T.H. & CHEN, C.J. (1989). Dose-response relation between arsenic concentration in well water and mortality from cancers and vascular diseases. *Am. J. Epidemiol.*, **130**, 1123.

YANG, G. & CHEN, C.W. (1991). A stochastic two-stage carcinogenesis model: a new approach to computing the probability of observing tumor in animal bioassays. *Math. Biosci.*, **104**, 247.